# Scaling human liver microphysiological systems: implementing a higher-throughput liver acinus microphysiological system platform

**DOI:** 10.3389/ebm.2026.11038

**Published:** 2026-05-22

**Authors:** Dillon C. Gavlock, Michael W. Castiglione, Allen Wang, Mahboubeh Varmazyad, Lawrence A. Vernetti, Mark E. Schurdak, D. Lansing Taylor, Jacquelyn A. Brown, Mark T. Miedel

**Affiliations:** 1 Organ Pathobiology and Therapeutic Institute, University of Pittsburgh, Pittsburgh, PA, United States; 2 Department of Computational and System Biology, School of Medicine, University of Pittsburgh, Pittsburgh, PA, United States; 3 Pittsburgh Liver Research Center, University of Pittsburgh, Pittsburgh, PA, United States; 4 Department of Pharmacology and Chemical Biology, University of Pittsburgh, Pittsburgh, PA, United States

**Keywords:** *in vitro* liver model, liver-on-a-chip, metabolic dysfunction–associated steatotic liver disease, microphysiological systems, new approach methodologies

## Abstract

The advancement in the use of all-human high content microphysiological systems (MPS) has enabled better *in vitro* modeling of liver function and disease progression as well as drug efficacy, metabolism and toxicity (ADME-Tox) testing. However, a continuing need in liver MPS development is balancing throughput without loss of the high-content biological complexity required for physiologically relevant modeling. Here, we present a scalable version of our well-established liver acinus microphysiological system (LAMPS). This higher-throughput format (ht-LAMPS) is designed to recapitulate the physiological complexity of the standard single-chamber LAMPS system while increasing experimental capacity through a seven-chamber microfluidic design. The ht-LAMPS is constructed using the same four key liver cell types as the LAMPS: primary hepatocytes and liver sinusoidal endothelial cells (LSECs) as well as Kupffer-like cells (THP-1) and hepatic stellate cells (LX-2). It recapitulates key physiological characteristics previously established in the LAMPS platform, including oxygen zonation–dependent liver phenotypes including model viability, secretion of functional and cytotoxicity markers, mitochondrial activity, and lipid accumulation, demonstrating reproducibility in the ht-LAMPS format. Finally, we also demonstrate that the ht-LAMPS model recapitulates key phenotypes associated with the progression of metabolic dysfunction–associated steatotic liver disease (MASLD), including increased steatosis and elevated production of inflammatory cytokines and profibrotic markers using our established MASLD media formulations. Overall, by increasing throughput while maintaining key high-content biological features of the LAMPS, ht-LAMPS provides a scalable platform for investigating liver function, modeling disease progression, and enabling downstream drug testing in MASLD and other liver-related conditions.

## Impact statement

Developing more scalable human liver microphysiological systems while retaining biological complexity is essential for improving in vitro modeling of liver disease and therapeutic response. Here, we build upon our well-established liver acinus microphysiological system (LAMPS) to create a higher-throughput LAMPS (ht-LAMPS) platform that recapitulates key high-content physiological features of the original model while enabling the modeling of several key phenotypes of metabolic dysfunction- associated steatotic liver disease (MASLD) progression. This approach supports more scalable studies of liver function, disease progression, and drug responses, advancing the translational relevance of in vitro liver models.

## Introduction

An important component of the FDA Modernization Act 2.0 is the elimination of the requirement for animal testing in the drug development process, allowing non-animal experimental data to be used for the evaluation of drug safety and efficacy [1, 2]. The goals of this change are to accelerate the drug development process and to reduce animal testing by promoting the use of non-animal experimental model systems. In further support of this shift, the National Institutes of Health (NIH) also recently announced that it will no longer award funding to grant proposals that rely solely on animal testing studies [3]. Thus, there is increased emphasis on implementing new approach methodologies (NAMs), human-relevant experimental and computational systems designed to replace or reduce animal testing while improving physiological relevance [4, 5]. NAMs include both complex *in vitro* models and *in silico* platforms such as organoids, spheroids, microphysiological systems (MPS), 3D bioprinting, and computational modeling approaches for modeling normal and pathological biological processes in a human-relevant context [6–8]. Human *in vitro* experimental models span a broad range of experimental throughput and biomimetic structure and functionality, including static 2D monocultures, static 3D spheroids and organoids, organoids in MPS, and both single organ and multi-organ coupled MPS [9, 10]. Biomimetic MPS are fluidic devices designed to reproduce key structural and spatial relationships among cells, physiological gradients, matrix environments, mechanical cues, and immune and neural interactions to approximate *in vivo* tissue and organ function [11].

Given the critical role of the liver in metabolism, drug-induced toxicity, and liver disease, numerous liver MPS platforms have been developed by academia and industry to model normal and pathological liver physiology, spanning a wide range of experimental throughput and biological complexity [11–29]. The unique organization of the liver sinusoid creates oxygen and metabolic gradients that drive zone-dependent hepatocyte functions [30–34]. Liver MPS platforms have therefore evolved to incorporate key aspects of liver architecture and function, enabling more biologically relevant modeling of both healthy and diseased liver physiology [10–18, 20–26, 35].

**FIGURE 1:**
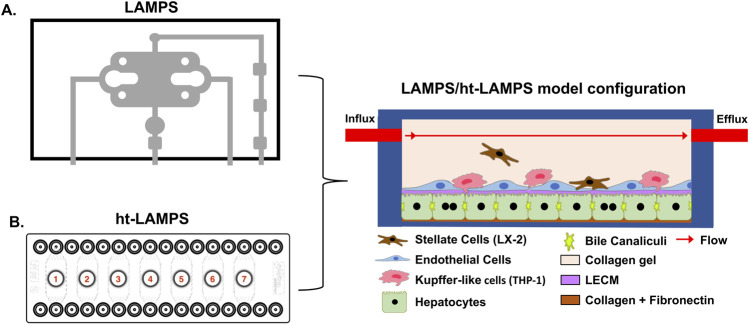
The LAMPS and higher throughput LAMPS (ht-LAMPS) platform overview. **(A)** Schematic diagram of the single chamber, PDMS-based Liver Acinus Microphysiological System (LAMPS) with glass bottom culture area. **(B)** Schematic of the higher throughput polystyrene based ht-LAMPS with 7 independent culture chambers. The two devices are constructed with four liver cell types (primary hepatocytes and liver sinusoidal endothelial cells (LSECS), as well as immortalized cell lines for hepatic stellate cells (LX-2) and Kupffer-like cells (THP-1). Both devices are maintained under continuous flow and can be maintained at various oxygen tensions corresponding to different liver zones. Representative 3D visualization of the multicellular architecture is provided in Supplementary Figure 1.

We have implemented a structured, biomimetic liver MPS platform, the liver acinus microphysiological system (LAMPS) (Figure 1) that is constructed with four key human liver cell types including hepatocytes, hepatic stellate cells (LX-2), liver sinusoidal endothelial cells (LSECs), and Kupffer-like cells (THP-1). The LAMPS can be constructed with multiple model configurations including a hybrid configuration consisting of both primary cells and immortalized cell lines, all-primary cells, and induced pluripotent stem cell (iPSC)-derived liver cells [20, 36–39]. The LAMPS is constructed through a combination of sequential cell layering and cell-to-cell self-organization between the 4 liver cell types and uses a single-chamber microfluidic design that supports sustained perfusion at controlled flow rates [37, 40]. This configuration enables maintenance of differential flow conditions that approximate the oxygen tensions characteristic of distinct liver zones (e.g., Zone 1 and Zone 3), allowing zone-dependent hepatocyte functions to be modeled within the same device [20, 37, 40]. The LAMPS includes a large set of phenotypic and molecular, secretome and fixed-endpoint readouts [20, 38, 39] and a database to capture and store the data and experimental metadata, as well as tools to analyze and model the data [41, 42]. A standard approach, the Pittsburgh Reproducibility Protocol (PReP), was developed that uses a set of common statistical metrics in a novel workflow to evaluate intra- and inter-study reproducibility of MPS performance across metrics [43].

The LAMPS has been validated by the National Center for Advancing Translational Sciences (NCATS) funded Tissue Chip Testing Centers (TCTC) and is being qualified as a drug development tool for hepatic clearance and toxicity in collaboration with the FDA as part of the Translational Centers for MPS (TraCe-MPS) program [37, 44, 45]. In addition, the LAMPS is also used as a disease progression and drug testing platform for metabolic dysfunction-associated steatotic liver disease (MASLD) [20, 36, 38, 39], to model the liver tumor microenvironment [46], and to evaluate biologics-induced liver injury [47].

As described above, human *in vitro* experimental models span a continuum of experimental throughput and biomimetic structure and functionality. There remains a need to further develop higher-throughput liver MPS that preserve biological complexity while supporting diverse experimental applications. Although the LAMPS exhibits high model complexity, its current single-chamber configuration limits overall experimental throughput. To address this challenge, we adapted the LAMPS to a commercially available seven-chamber microfluidic device format, thereby increasing throughput while maintaining model complexity. This ht-LAMPS enables more scalable, high-content modeling of both normal and diseased liver physiology for downstream mechanistic and drug response studies. In this report, we demonstrate the reproducibility of ht-LAMPS and illustrate its performance using metabolic dysfunction–associated steatotic liver disease (MASLD) as a representative disease context.

## Materials and methods

### Cell sources and initial culture

The following lot of commercially available primary human hepatocytes was used in this study: hu8391 (ThermoFisher Scientific). Human liver sinusoidal endothelial cells (LSECs) were purchased from LifeNet Health (NPC-AD-LEC-P1). The THP-1 human monoblast cell line (ATCC, TIB-202) was used to generate Kupffer-like cells and was pretreated 48 h before seeding with 200 ng/mL phorbol 12-myristate 13-acetate (PMA; MilliporeSIgma, 524400) to induce macrophage-like differentiation and inhibit proliferation. Human hepatic stellate cells (LX-2) were obtained from MilliporeSIgma (SCC064). Cell culture conditions were as follows: LSECs: cultured in endothelial cell basal medium-2 (EBM-2; Lonza, CC-3162). THP-1 cells: maintained in suspension in RPMI 1640 (Cytiva, SH30096.FS) with 10% fetal bovine serum (FBS; Corning, MT35010CV), 100 μg/mL penicillin-streptomycin (Cytiva, SV30010), and 2 mM L-glutamine (Cytiva, SH30034.01). LX-2 cells: cultured in DMEM (ThermoFisher Scientific, 11965118) supplemented with 2% FBS, 100 U/mL penicillin, and 100 μg/mL streptomycin [20, 36–38, 40, 46].

### LAMPS assembly and maintenance

ht-LAMPS were assembled and maintained as previously described [20, 36–38, 40, 46] except using the Fluidic 557 Reaction Chamber Chip (Microfluidic ChipShop #557). The ht-LAMPS were constructed using four liver cell types at the following cell densities: primary cryopreserved human hepatocytes (ThermoFisher lot hu8391 2.75 × 10^6^ cells/mL), primary liver sinusoidal endothelial cells (LifeNet Health; LSECs 1.5 × 10^6^ cells/mL), and THP-1 (ATCC; 0.4 × 10^6^ cells/mL) and LX-2 (MilliporeSigma; 0.2 × 10^6^ cells/mL). The percentages of hepatocytes (56%), THP-1 (18%), LSEC (22%), and LX-2 (4%) cells are consistent with the scaling used in our previously published models. The interior of the devices were coated for 2 h at RT or overnight at 4 °C with 100 μg/mL bovine fibronectin (MilliporeSigma, F1141) and 150 μg/mL rat tail collagen (Corning, 354249) in PBS prior to cell seeding. For all steps involving injection of media and/or cell suspensions into LAMPS devices, 90–100 μL per device was used to ensure complete filling of fluidic pathways and chamber. The devices were then overlayed with 1.5 mg/mL rat tail collagen I (Corning) and maintained with the perfusion of different conditions for 8 days at a flow rate of 5 μL/h to recapitulate zone 3 oxygen tension [38, 40].

### Media formulation; normal fasting, early metabolic syndrome, and late metabolic syndrome media

MASLD disease phenotypes in LAMPS were driven using defined media formulations to mimic disease progression from normal fasting (NF) to early metabolic syndrome (EMS) and then late metabolic syndrome (LMS) for 8 days [20, 38]. These media formulations were developed using glucose-free Williams E base medium (ThermoFisher, ME18082L1) supplemented with physiologically relevant levels of glucose (MilliporeSIgma, G8644), insulin (ThermoFisher, 12585014), and glucagon (MilliporeSIgma and molecular drivers of fibrosis including TGF-β1 (ThermoFisher, PHG9214) and lipopolysaccharide (MilliporeSIgma, L2654). The specific concentrations of all media components used for the NF, EMS, and LMS formulations are provided in Supplementary Table 1.

### LipidTOX staining and immunofluorescence

Devices were stained using established protocols [8, 20, 37]. Cells were fixed with 4% paraformaldehyde (ThermoFisher Scientific, AA433689M) in PBS for 30 min, followed by two washes with PBS. For lipid staining, LipidTOX Deep Red Neutral Lipid Stain (1:500; Invitrogen, H34477) was perfused into devices and incubated overnight at 4 °C. For immunofluorescence, chambers were labeled with either rabbit polyclonal anti-cytokeratin 18 (CK-18) antibody (ThermoFisher Scientific, PA5-14263; 1:200) and mouse monoclonal anti-α-smooth muscle actin (αSMA) antibody (Sigma-Aldrich, A2547; 1:100), or with rabbit polyclonal anti-collagen 1A1 (COL1A1) antibody (Proteintech, 14695; 1:100), and incubated overnight at 4 °C. The following day, devices were washed twice with PBS and then incubated for 2 h at room temperature with Alexa Fluor goat anti-rabbit 488 (1:250; Invitrogen, A-11029) and Alexa Fluor goat anti-mouse 568 (1:250; Invitrogen, A-11004) secondary antibodies, along with Hoechst (5 μg/mL; Invitrogen, H1399). Devices were washed three times with PBS prior to imaging.

### TMRE and calcein AM staining

Mitochondrial membrane potential was assessed in live LAMPS using tetramethylrhodamine ethyl ester (TMRE; ThermoFisher Scientific, Cat. No. T669). Devices were equilibrated in pre-warmed culture medium containing 100 nM TMRE for 15 min at 37 °C. Following incubation, channels were gently rinsed with fresh medium to remove excess dye and immediately imaged under live-cell conditions using confocal fluorescence microscopy (ex/em = 549/575 nm). For endpoint live cell imaging, cultures were stained with Calcein AM (ThermoFisher C1430) and Hoechst nuclear dye (ThermoFisher 62249). A 1 mg/mL Calcein AM stock solution was prepared in DMSO and diluted 1:1000 in normal fasting medium to yield a final concentration of approximately 1 μM, together with Hoechst (1:2000; ∼9 µM final). Following chip disconnection, 50 µL of staining solution was applied to both the inlet and outlet, and chips were incubated for 45 min at 37 °C. Imaging was performed at ×10 magnification using the FITC (Calcein AM) and DAPI (Hoechst) channels.

### Confocal imaging and analysis

Imaging was conducted on the Phenix High-Content Imaging system (Revvity) using a 20×/0.4 NA air objective. Z-stacks covering 35 μm depth (5 μm steps) were acquired across 7 × 15 adjacent fields (total area: 44 mm^2^; 105 total fields per chamber). All images were collected using consistent exposure and laser power settings to maintain signal intensity within 50–90% of the dynamic range. Image analysis was performed using Harmony software (Revvity, v5.1).

### Quantification of calcein-positive cells

Live cell quantification was performed on Day 8 using an automated image analysis workflow implemented in Harmony (PerkinElmer). The analysis utilized the DAPI (nuclear) (ex: 405/em: 435–480) and calcein (live cell) (ex: 488/em: 500–550) fluorescence channels to identify total and live cell populations. Nuclei were segmented using the “Find Nuclei” block set to method M in the Harmony 5.1 software, and calcein-positive regions were detected using optimized intensity thresholding within the “Find Image Region” block. Then nuclei not overlapping with calcein-positive regions were excluded using the “Select Population” block. The percentage of live cells was determined as:

$$\%\text{ Calcein}‐\text{positive cells}=\left(\frac{Calcein-positive\;nuclei}{Total\;Hoechst-positive\;nuclei}\right)\times 100$$

### Mitochondrial activity quantification (TMRE)

Mitochondrial membrane potential was quantified on Day 8 from TMRE fluorescence images using automated segmentation and intensity-based analysis. Nuclei were first identified using the DAPI channel using the “Find Nuclei” block in the Harmony 5.1 software, and the corresponding perinuclear cytoplasmic regions were defined for each cell using the “Find Surrounding Region” block on method A. TMRE-positive (ex: 561/em: 570–630) signal intensity and area were measured within these regions, and integrated density was calculated as the product of TMRE-positive area and mean fluorescence intensity to represent mitochondrial activity per cell.

### LipidTOX staining and quantification

Steatosis was quantified on Day 8 from LipidTOX-stained images using automated segmentation and object detection workflows (Harmony). Nuclei were identified from the DAPI channel, and lipid-positive regions were segmented based on LipidTox fluorescence (ex: 640/em: 650–760). Individual lipid droplets between 5 and 30 μm in diameter were detected and counted. Lipid accumulation was report both in terms of the average number of lipid droplets per cell (total droplets divided by total nuclei) and the mean lipid-positive area per imaging field.

### Efflux collection and functional analysis

Albumin (ALB), blood urea nitrogen (BUN), lactate dehydrogenase (LDH), and pro-collagen Iα1 (COL 1A1) levels were assessed as previously described [20, 36, 37, 46]. Efflux from the LAMPS was collected on days 2, 4, 6, and 8 for analysis. ALB levels were quantified using an enzyme-linked immunosorbent assay (ELISA) on 1:100 efflux dilutions with commercial antibodies (Bethyl Laboratories, A80-129A and A80-129P) and an ELISA accessory kit (Bethyl Laboratories, E101), with a human albumin standard prepared in-house (MilliporeSigma, 126658). Secreted COL 1A1 was quantified using the human pro-collagen Iα1 ELISA kit (R&D Systems, cat. no. DY6220-05) in a 1:50 efflux dilution. BUN levels were determined using the Stanbio BUN Liquid Reagent for Diagnostic Set (Stanbio Laboratory, cat. no. SB-0580–250), while LDH levels were assessed using the CytoTox 96 Non-Radioactive Cytotoxicity Assay (Promega, cat. no. G1780). The BUN and LDH assays were adapted to a 384-well microplate format without efflux dilution.

### Multiplex immunoassays

The levels of IL-6, IL-8, and MCP-1 were determined in efflux collected on Day 8 using a custom version of the Human XL Cytokine Performance Panel (R&D systems). Assays were completed according to the manufacturer’s instructions at The University of Pittsburgh Cancer Proteomics Facility Luminex® Core Laboratory on the xMAP platform.

### Oxygen tension modeling and validation

Oxygen tension was quantified in the ht-LAMPS as previously described [10, 16, 40] and validated via ratio imaging using oxygen-sensitive and oxygen-insensitive fluorescent beads. Oxygen supply in the sealed microfluidic LAMPS device was modeled to determine the flow rate required to achieve target oxygen tension. Using a 3D COMSOL Multiphysics model (v5.2a) [10, 16, 40], flow was described by Brinkman equations through the ECM and cell layer, and oxygen transport/consumption was modeled using the “transport of diluted species” module with a maximum primary hepatocyte OCR of 450 pmol s^−1^ 10^6^ cells^−1^. The model predicted that 5 µL h^−1^ would maintain 6–8% oxygen tension, which was confirmed experimentally with oxygen-sensitive beads (Bangs Laboratories). PdTFPP-loaded oxygen-sensitive beads (ex/em 405/706 nm) and polystyrene oxygen-insensitive beads (Bangs Laboratories) (ex/em 405/455 nm) were obtained from Bangs Laboratories and prepared as previously described [10, 16, 40]. Validation was performed in simplified ht-LAMPS containing only hepatocytes and the bead mixture. This configuration was used to isolate the dominant oxygen-consuming hepatocyte cell population [48, 49] and enable controlled calibration of the bead-based oxygen measurements. At the seeding ratios used here, hepatocytes are the primary drivers of oxygen consumption in the system, and prior experimental and computational studies support hepatocyte-driven oxygen gradients under these conditions. Chips were pre-treated with ECM overnight, seeded with hepatocytes, and incubated for 4 h. The bead mixture were added in LECM and allowed to settle overnight. Chips were overlaid with collagen, polymerized after 4 h, and perfused at 5 μL/h in NFM (18% dissolved oxygen) for 7 days. Imaging was performed using the IN-Cell Analyzer 6000. Calibration was done using the same chips: Max O_2_ (18%): Achieved by increasing flow to 50 μL/h. Min O_2_ (0%): Achieved by adding 200 μg/mL glucose oxidase (MilliporeSigma) to the media. Final oxygen tension was determined as ratio between the oxygen sensitive and insensitive beads [40].

### Statistical analysis

For comparisons between two groups, the student’s t-test with Welch’s Correction was used, while multigroup comparisons used a One-Way ANOVA with Tukey’s test for multiple comparisons. For all statistical analyses, a p-value of 0.05 was used as criteria for statistical significance. Reproducibility analysis was assessed using the Eve Analytics™ platform following the Pittsburgh Reproducibility Protocol (PReP) [43]. Intraclass correlation coefficient (ICC) was used to assess both intra- and inter-study reproducibility over time for the efflux metrics. Model performance reproducibility was classified as: Excellent: ICC ≥0.8 Acceptable: 0.2 ≤ ICC <0.8 Poor: ICC <0.2. The coefficient of variation (CV) was used to assess the reproducibility of the single time point measurements model performance reproducibility was classified as: Excellent: CV ≤ 5%; Acceptable: 5% ≤ CV < 20%; Poor: CV ≥ 20%.

## Results

### Development of a higher-throughput liver acinus microphysiological system (ht-LAMPS)

To increase experimental throughput while preserving the biological complexity of the original LAMPS platform, the single-chamber device (Figure 1A) was adapted to a commercially available seven-chamber microfluidic format (Figure 1B). The resulting higher-throughput LAMPS (ht-LAMPS) maintains the same layered, multicellular architecture as the original LAMPS, as confirmed by 3D imaging of cell-type distribution within the device using CellTracker labeling (Supplementary Figure 1), and supports continuous perfusion under flow conditions corresponding to Zone 1 and Zone 3 oxygen tensions. Each chamber functions as an independent model, enabling parallel experimental conditions within a single device. This configuration increases experimental capacity while using the same cell composition, media formulations, and perfusion parameters established for the standard LAMPS platform.

### Oxygen zonation modeling and validation

**FIGURE 2:**
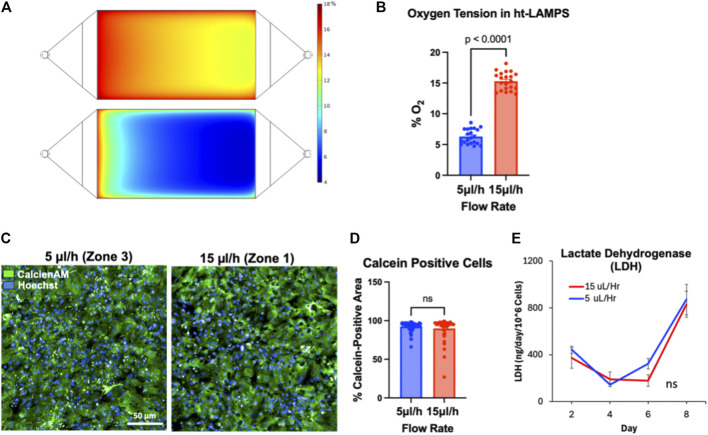
Validation of oxygen tension, viability, and cytotoxicity in ht-LAMPS. **(A)** COMSOL modeling predicted oxygen tension profiles in ht-LAMPS at perfusion rates of 5 µL/h (Zone 3) and 15 µL/h (Zone 1). **(B)** Experimental quantification using oxygen-sensitive beads in hepatocyte-only ht-LAMPS confirmed significantly higher oxygen levels at 15 µL/h (∼15% O_2_) compared with 5 µL/h (∼6% O_2_) (p < 0.0001, n = 21 chambers). **(C)** Representative fluorescence images of ht-LAMPS after 8 days under flow show calcein-positive viable cells and Hoechst-labeled nuclei (20x; scale bar = 50 µm). **(D)** Quantification on Day 8 of calcein-positive area demonstrated no significant difference in model viability between Zone 1– and Zone 3–like flow conditions after 8 days in culture (n = 21 chambers). **(E)** Lactate dehydrogenase (LDH) secretion did not differ significantly between zones, indicating comparable cytotoxicity under both oxygen conditions (one-way ANOVA; n = 21 chambers). Data are presented as mean ± SEM.

A key functional feature of the LAMPS platform is the ability to reproduce hepatic zonation through controlled oxygen tension through defined media flow conditions [19, 31, 46]. Computer Solutions (COMSOL) modeling was used to estimate the perfusion rates required to achieve zone-specific oxygen levels in the ht-LAMPS platform (Figure 2A) [19, 31, 46]. Liver zonation was induced in ht-LAMPS by modulating oxygen tension through controlled perfusion rates. COMSOL simulations predicted that a flow rate of 5 µL/h produces zone 3–like oxygen tension (∼6% O_2_), whereas 15 µL/h yields zone 1–like oxygen tension (∼15% O_2_) (Figure 2A).

These predictions were validated experimentally using oxygen-sensitive beads in hepatocyte-containing chips, which serve as a simplified model for the full system given the dominant contribution of hepatocytes to oxygen consumption [16, 40, 48, 49], with oxygen levels quantified by ratiometric imaging based on the ratio of oxygen-sensitive to oxygen-insensitive fluorescence intensity (Figure 2B; p < 0.0001, n = 21chambers) [19, 31, 46]. In addition, ht-LAMPS model viability under both oxygen conditions was comparable after 8 days of culture under flow, as assessed by Calcein AM staining, with no significant difference in the percentage of Calcein-positive cells (Figures 2C,D; n.s., n = 21 chambers) nor in lactate dehydrogenase (LDH) secretion, a marker of cytotoxicity (Figure 2E; n.s., n = 21chambers). Together, these data confirm that stable, zone-specific oxygen conditions can be established in ht-LAMPS without adverse effects on model viability or cytotoxicity.

### Functional validation of hepatic zonation

**FIGURE 3:**
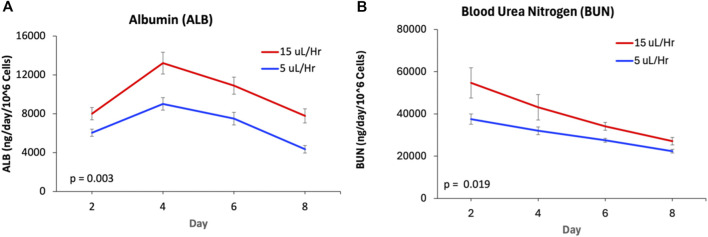
Zone-specific differences in albumin and blood urea nitrogen secretion are recapitulated in ht-LAMPS. Albumin (ALB) and blood urea nitrogen (BUN) secretion were quantified over an 8-day time course in ht-LAMPS maintained under perfusion rates of 15 µL/h (Zone 1) or 5 µL/h (Zone 3). ht-LAMPS maintained at 15 µL/h exhibited significantly higher ALB **(A)** and BUN **(B)** secretion compared with those maintained at 5 µL/h (one-way ANOVA: ALB, p = 0.003, n = 21 chambers; BUN, p = 0.019, n = 21 chambers). Data are presented as mean ± SEM.

Oxygen gradients across the liver lobule drive zonation-dependent differences in hepatocyte function and protein secretion [16, 31, 40, 50]. To evaluate whether the ht-LAMPS recapitulates zone-specific hepatocyte functions, effluent media were collected and analyzed for albumin and urea nitrogen as indicators of hepatocyte secretory and metabolic activity. Consistent with clinical observations [51], albumin secretion was significantly higher in Zone 1 than in Zone 3 throughout the experimental time course (Days 2–8) (Figure 3A; p = 0.003, n = 21chambers). Similarly, urea nitrogen secretion was significantly greater in Zone 1 ht-LAMPS compared with Zone 3 over the same period (Figure 3B; p = 0.019, n = 21 chambers), consistent with zonation-dependent secretion patterns previously reported for *in vivo* human liver and the LAMPS platform [20, 38, 40, 52].

**FIGURE 4:**
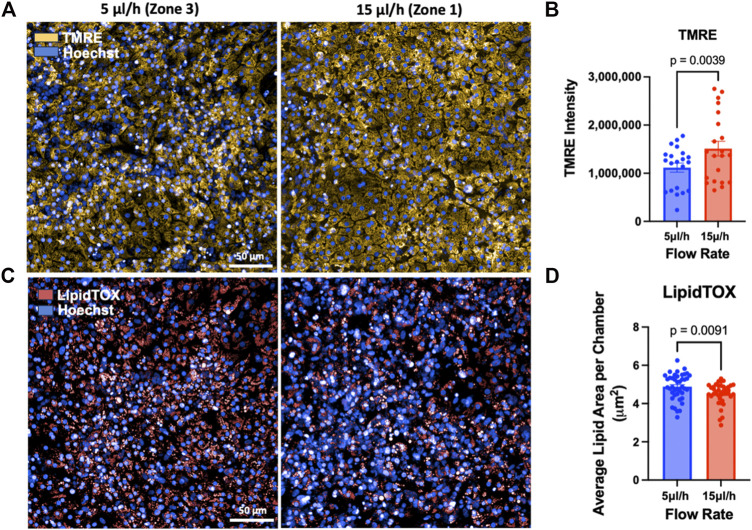
Zone-specific differences in mitochondrial activity and steatosis in ht-LAMPS. Representative Day 8 fluorescence images **(A)** and quantification **(B)** of tetramethylrhodamine ethyl ester (TMRE) staining demonstrate significantly higher mitochondrial activity in Zone 1–like ht-LAMPS compared with Zone 3–like conditions (20×; scale bar = 50 μm; p = 0.0039, n = 21 chambers). Representative images **(C)** and quantification **(D)** of LipidTOX staining demonstrate significantly greater lipid accumulation in Zone 3–like ht-LAMPS compared with Zone 1–like conditions (20x; scale bar = 50 μm; p = 0.0091, n = 21 chambers). Data are presented as mean ± SEM.

To further assess zonation-dependent phenotypes, mitochondrial activity and steatosis, which are known to differ by liver zone [24, 46, 53], were quantified in ht-LAMPS maintained at Zone 1 or Zone 3 flow rates for 8 days. Tetramethylrhodamine ethyl ester (TMRE) staining intensity was significantly higher in Zone 1 ht-LAMPS compared with Zone 3 on Day 8 (Figures 4A,B; p = 0.0039, n = 21 chambers), consistent with increased mitochondrial activity in Zone 1 [40]. In contrast, lipid accumulation, assessed using the neutral lipid dye LipidTOX, was significantly greater in Zone 3 ht-LAMPS than in Zone 1 on Day 8 (Figures 4C,D; p = 0.0091, n = 21 chambers), consistent with known zone-specific patterns of hepatic steatosis and with our prior LAMPS-based studies [20, 40, 54]. Together, these results demonstrate that ht-LAMPS reproduces physiologically relevant, zone-specific functional phenotypes consistent with those previously observed in the LAMPS platform [20, 40].

### ht-LAMPS demonstrates reproducibility across multiple assay metrics

Reproducibility was assessed in the ht-LAMPS for the experimental metrics used to generate Figures 2–4 using the Pittsburgh Reproducibility Protocol (PReP) [43] in combination with the EveAnalytics™ platform [41, 42]. Multi–time point metrics (albumin, urea nitrogen, and lactate dehydrogenase as well as single–time point metrics collected on Day 8 (Calcein AM, TMRE, and LipidTOX) were analyzed to quantify both intra- and inter-study reproducibility (Table 1). For multi–time point metrics, intraclass correlation coefficients (ICC) indicated strong intra- and inter-study reproducibility for albumin and urea nitrogen secretion. In contrast, LDH exhibited poor inter-study reproducibility overall, although intra-study reproducibility was generally acceptable except for Study 2. For single–time point metrics (Day 8), coefficients of variation (CV) indicated high inter-study reproducibility for Calcein AM, TMRE, and LipidTOX measurements. Calcein AM and LipidTOX also showed high intra-study reproducibility, while TMRE exhibited greater variability within individual studies. Together, these results indicate that ht-LAMPS exhibits reproducible performance for several key functional and phenotypic assay metrics, while also revealing greater variability in selected readouts, including LDH and TMRE. Notably, TMRE measurements showed higher intra-study variability but consistent directional trends across studies, whereas LDH exhibited variability across both intra- and inter-study comparisons. These findings highlight both the strengths of the platform and areas where additional assay optimization may be beneficial.

**TABLE 1:** ht-LAMPS demonstrate intra- and inter-study reproducibility across multiple key metrics.

Metric	Flow rate (μL/hr)	Intra-study reproducibility											
		Study 1			Study 2			Study 3			Inter-study reproducibility		
		Metric	Value	Status	Metric	Value	Status	Metric	Value	Status	Metric	Value	Status
Albumin	15	ICC	0.35	Acceptable	ICC	0.29	Acceptable	ICC	0.42	Acceptable	ICC	0.58	Acceptable
	5	ICC	0.2	Acceptable	ICC	0.55	Acceptable	ICC	0.49	Acceptable	ICC	0.83	Excellent
BUN	15	ICC	0.07	Poor	ICC	0.64	Acceptable	ICC	0.44	Acceptable	ICC	0.86	Excellent
	5	ICC	0.26	Acceptable	ICC	0.63	Acceptable	ICC	0.39	Acceptable	ICC	0.81	Excellent
LDH	15	ICC	0.71	Acceptable	ICC	0.05	Poor	ICC	0.66	Acceptable	ICC	−0.05	Poor
	5	ICC	0.82	Excellent	ICC	0.61	Acceptable	ICC	0.5	Acceptable	ICC	0.11	Poor
Calcein	15	CV	7.11	Acceptable	CV	13.41	Acceptable	CV	3.37	Excellent	CV	1.17	Excellent
	5	CV	7.7	Acceptable	CV	2.54	Excellent	CV	4.66	Excellent	CV	1.68	Excellent
TMRE	15	CV	23.17	Poor	CV	22.6	Poor	CV	11.75	Acceptable	CV	15.7	Acceptable
	5	CV	66.35	Poor	CV	26.5	Poor	CV	51.21	Poor	CV	17.3	Acceptable
LipidTOX	15	CV	8.81	Acceptable	CV	14.47	Acceptable	CV	5.36	Acceptable	CV	3.623	Excellent
	5	CV	12.67	Acceptable	CV	7.94	Acceptable	CV	7.26	Acceptable	CV	13.03	Acceptable

For multi-time point measurements model reproducibility was classified as: Excellent: ICC ≥0.8; acceptable: 0.2 ≤ ICC <0.8; poor: ICC <0.2. For single time point measurements model reproducibility was classified as: Excellent: CV ≤ 5%; acceptable: 5% ≤ CV < 20%; poor: CV ≥ 20%.

To directly compare the performance of the ht-LAMPS with the previously published single-chamber LAMPS platform [20, 40, 42, 47], zonation-dependent functional metrics measured at Day 8 were compared across platforms using ht-LAMPS data generated in this study and single-chamber LAMPS datasets from prior studies (Supplementary Table 2; Supplementary Figure 2). Supplementary Figure 2 shows these comparisons, allowing for the visualization of the zonation-dependent trends wthin each platform. Ratios of Zone 3 to Zone 1 responses for albumin, urea nitrogen, mitochondrial activity (TMRE), and steatosis (LipidTOX) showed consistent directional trends between the two platforms. Across these metrics, ht-LAMPS recapitulated key zone-specific patterns previously observed in the single-chamber LAMPS, including higher metabolic activity in Zone 1 and increased steatosis in Zone 3. While these comparisons are not derived from fully matched head-to-head experiments, they indicate that scaling the LAMPS setup to a multi-chamber format recapitulates key zonation-dependent functional relationships for the metrics evaluated in this study.

### ht-LAMPS recapitulates key MASLD-associated phenotypes

We have previously developed and published a series of media formulations, normal fasting media (NF), early metabolic syndrome media (EMS), and late metabolic syndrome media (LMS), to experimentally model lifestyle-driven progression of MASLD (Supplementary Figure 1) [20, 38]. These formulations were designed based on clinical blood chemistries and have been used to characterize multiple MASLD-associated phenotypes, including steatosis, secretion of pro-inflammatory cytokines, and pro-collagen 1α1 (COL 1A1) in the LAMPS platform.

**FIGURE 5:**
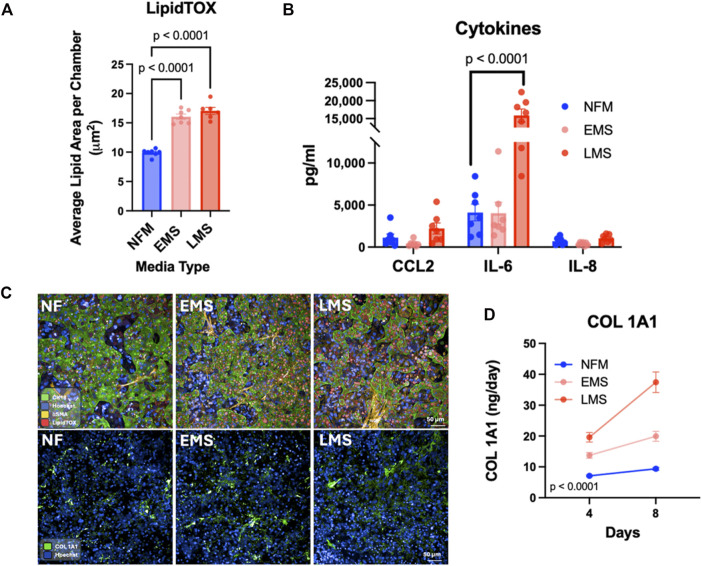
ht-LAMPS recapitulates key MASLD-associated phenotypes under disease-inducing media conditions. ht-LAMPS were maintained for 8 days under Zone 3 flow conditions and cultured in normal fasting (NF), early metabolic syndrome (EMS), or late metabolic syndrome (LMS) media formulations previously established to model MASLD progression in the LAMPS platform [20, 36, 38]. **(A)** Steatosis, assessed by LipidTOX staining, was significantly increased in both EMS- and LMS-treated ht-LAMPS compared with NF controls, indicating enhanced steatosis under disease-inducing conditions on Day 8 (p < 0.0001, n = 7 chambers). **(B)** Evaluation of inflammatory cytokine secretion showed increased levels across cytokines in the LMS condition compared with NF, with IL-6 exhibiting a statistically significant increase on Day 8 (p < 0.0001, n = 7 chambers). **(C)** Representative Day 8 immunofluorescence images demonstrating expression of LipidTOX and α-smooth muscle actin (αSMA; top panels) and collagen 1α1 (COL 1A1; bottom panels) in ht-LAMPS cultured under NF, EMS, and LMS conditions. In the top panels, hepatocytes are labeled with cytokeratin-18 (CK-18) as a marker of hepatocytes. Increased αSMA and COL 1A1 staining is observed under EMS and LMS conditions relative to NF, consistent with increased stellate cell–associated and fibrogenic marker expression. Nuclei are labeled with Hoechst. Scale bars = 50 µm. The spatial organization of hepatocytes (CK-18 and LipidTOX) and stellate cells (αSMA) in ht-LAMPS under these MASLD-inducing conditions is shown in Supplementary Figure 3, which illustrates both cell distribution and disease-associated changes in marker expression. **(D)** Secretion of the pro-fibrotic marker COL 1A1 was significantly elevated in EMS and LMS conditions relative to NF, with a stepwise increase from EMS to LMS on Day 8 (one-way ANOVA; p < 0.0001 for both EMS and LMS, n = 7 chambers). Data are presented as mean ± SEM.

To determine whether the ht-LAMPS recapitulates these MASLD-associated phenotypes, models were cultured in NF, EMS, and LMS media (Supplementary Figure 1) and evaluated on Day 8. Because MASLD-associated injury and lipid accumulation are most pronounced in the pericentral (Zone 3) region of the liver [53, 55], ht-LAMPS were maintained under Zone 3–like flow conditions, consistent with the experimental design used in our previous MASLD studies with the LAMPS platform [20, 36, 38]. Both EMS and LMS media induced significantly greater lipid accumulation compared with NF media (Figure 5A; p < 0.0001, n = 7 chambers). In parallel, secretion of inflammatory cytokines increased under disease-inducing conditions, with IL-6 showing a significant elevation in the LMS condition relative to NF (Figure 5B; p = 0.0001, n = 7 chambers). CCL2 and IL-8 also exhibited increasing trends in the LMS condition compared with NF (Figure 5B). These cytokines (IL-6, IL-8, and CCL2) were selected as representative inflammatory mediators based on their established roles in MASLD progression and hepatic inflammatory responses [56, 57], and their use in prior LAMPS studies [20, 36, 38]. In addition, secretion of the pro-fibrotic marker COL 1A1 increased in a stepwise manner from EMS to LMS (Figure 5D; p < 0.0001, n = 7 chambers). Complementing these efflux-based measurements, immunofluorescence imaging of ht-LAMPS (Figure 5C) demonstrated increased expression of α-smooth muscle actin (αSMA) and COL1A1 under EMS and LMS conditions relative to NF. These data provide cell-associated evidence of increased stellate cell–associated and fibrogenic marker expression under disease-inducing conditions.

To assess whether MASLD-associated phenotypes observed in ht-LAMPS are consistent with those previously reported in the single-chamber LAMPS [20, 36, 38], disease-response metrics measured at Day 8 were compared between platforms under matched media conditions (Supplementary Table 3). Both platforms exhibited similar increases in steatosis and secretion of the pro-fibrotic marker COL 1A1 in response to EMS and LMS media, indicating preserved induction of key metabolic and fibrotic MASLD progression phenotypes in the ht-LAMPS configuration. Cytokine responses were also broadly comparable between platforms under LMS conditions, with both systems showing increased IL-6 secretion and increasing trends in CCL2 and IL-8. While cytokine induction under EMS conditions was attenuated in ht-LAMPS relative to the single-chamber LAMPS, the overall pattern of MASLD-associated metabolic, fibrotic, and inflammatory responses was consistent between platforms.

## Discussion

In this study, we demonstrate that our previously established, high-content liver MPS, the liver acinus microphysiological system (LAMPS) [11, 20, 36–40, 46], can be adapted from a single-chamber format to a multi-chamber (7 chambers per chip) configuration while recapitulating its key physiological and functional characteristics. The higher-throughput LAMPS (ht-LAMPS), compared to the original single-chamber device, maintained controlled oxygen zonation, reproduced zone-dependent hepatocyte functions, and recapitulated key MASLD progression phenotypes, including steatosis, inflammatory cytokine secretion, and pro-fibrotic marker production. Across these readouts, ht-LAMPS exhibited reproducible performance for several key metrics (Table 1) and recapitulated key biological features previously observed in the single-chamber LAMPS platform for the metrics evaluated in this study [20, 37, 38, 40, 45]. Despite differences in device design, material properties, and chamber configuration, the same underlying biological system produced broadly similar functional, metabolic, and disease-associated phenotypes in both the single-chamber LAMPS and the seven-chamber ht-LAMPS formats. The single-chamber LAMPS employs a square PDMS-based chamber, whereas the ht-LAMPS utilizes a polystyrene device with trapezoidal prism–shaped chambers, and the two platforms also differ substantially in chamber height (approximately 170 µm versus ∼700 μm, respectively). Despite these differences in geometry, material composition, and chamber dimensions, controlled oxygen zonation and zone-dependent functional outputs were maintained under the conditions evaluated. Direct cross-platform comparisons demonstrated similar zonation-dependent functional patterns across both platforms, as assessed by metabolic activity, mitochondrial function, and steatosis (Figures 2–4; Supplementary Table 2). Comparisons of MASLD-associated phenotypes further showed that steatosis and pro-fibrotic responses between platforms were similar (Figure 5; Supplementary Table 3), with only modest differences in inflammatory cytokine responses observed under early disease (EMS) conditions. Together, these findings indicate that the biological performance of the LAMPS model is recapitulated across differences in device architecture and material properties, supporting its adaptability for use across distinct microfluidic formats.

Recent advances in liver MPS have increasingly emphasized incorporation of vascular structures, immune components, and multicellular spheroid/organoid designs to better recapitulate aspects of native liver physiology and disease [27–29]. These platforms demonstrate the ability to capture complex cell–cell interactions, including vascular perfusion and immune cell recruitment, representing important advances in modeling higher-order liver physiology. In this context, ht-LAMPS is designed as a complementary approach that prioritizes controlled zonation, reproducible performance, and longitudinal functional readouts. The platform enables defined and tunable oxygen and flow conditions that reproducibly generate zone-specific phenotypes. In addition, the relatively low perfusion rates support collection of concentrated effluent, enabling sensitive longitudinal analysis of secreted factors, which can be more challenging in higher-flow or highly vascularized systems. Accordingly, ht-LAMPS occupies a distinct position within the current MPS landscape, bridging simplified high-throughput systems and more complex vascularized models, and is particularly well-suited for applications requiring controlled perturbation of metabolic and zonation-dependent processes.

The primary technical advance of this study is a practical increase in experimental throughput while preserving model complexity. Adaptation of LAMPS to a multi-chamber format enables an approximately four-fold increase in experimental capacity relative to the original single-chamber system, while maintaining the established biological architecture, cell composition, and experimental framework of the platform. Across the broader landscape of MPS models, platform selection reflects a balance between throughput and biological complexity that is driven by the specific question and context of use. While higher-throughput liver MPS platforms are employed for various applications [14, 58–62], these often rely on simplified tissue architectures or emphasize different aspects of physiological complexity and control. In this context, the ht-LAMPS represents a meaningful increase in capacity within a human-relevant liver MPS that retains the complexity required for zonated liver function, disease modeling, and downstream mechanistic and therapeutic studies.

The development of ht-LAMPS also aligns with the increasing emphasis on human-based experimental systems in biomedical research. Recent policy and funding initiatives, including the FDA Modernization Act 2.0 and updated NIH guidance, underscore the need for experimentally tractable human-relevant models that complement traditional animal studies and improve the translational relevance of preclinical research [1, 3, 6, 63]. By preserving physiological complexity while increasing experimental capacity, ht-LAMPS contributes to this evolving landscape by providing a human liver MPS with enhanced experimental capacity suitable for disease-relevant mechanistic studies and therapeutic evaluation.

Although the ht-LAMPS represents a meaningful step forward, additional scalability remains an important area for future development of this platform. In addition, future studies will evaluate how varying the number of active chambers within a device influences flow distribution, oxygen gradients, and assay performance, to further define the operating range and scalability of the platform. Further gains in overall model scalability may be achieved through device-level refinements, including increasing chamber number per chip without compromising assay sensitivity, implementing fluidic designs that evenly distribute flow across parallel chambers from a single inlet, miniaturizing culture volumes to enable higher-density layouts, integrating multiplexed real-time sensors to reduce manual sampling, and adopting device formats compatible with the robotic liquid-handling systems used in high-throughput laboratories. In parallel, advances in automation, including bioprinting-based approaches for controlled cell and matrix deposition, could reduce operator time and improve reproducibility; however, because ht-LAMPS is implemented in a closed-chip format, these approaches will need to be adapted for enclosed microfluidic environments. These developments must balance increased scalability with preservation of the spatial organization, flow control, and multicellular interactions that underpin the biological performance of the LAMPS model.

The observed differences in inflammatory cytokine responses between the single-chamber LAMPS and ht-LAMPS under EMS conditions highlight the value of direct cross-platform comparisons. While cytokine responses under LMS conditions were broadly similar between formats, the attenuated response under EMS conditions may reflect differences in the experimental platforms or biological variability, including cell sourcing. These findings motivate future studies to more systematically characterize how experimental design parameters, such as disease stage, chamber format, and cell source, influence inflammatory readouts across device formats.

In summary, this work establishes ht-LAMPS as a more scalable extension of our validated LAMPS platform that recapitulates key physiological and functional characteristics while increasing experimental capacity. The ht-LAMPS maintained controlled oxygen zonation, reproduced zone-dependent hepatocyte functions, and exhibited reproducible performance consistent with the original single-chamber LAMPS. In addition, ht-LAMPS recapitulated core MASLD progression phenotypes, including steatosis, inflammatory cytokine secretion, and pro-fibrotic marker production, supporting its use for downstream mechanistic studies and drug development applications.

## Data Availability

The datasets generated and analyzed during the current study are available from the corresponding author on reasonable request. In addition, the data from experimental studies performed in this work are available at: https://eveanalytics.com/accounts/login/?next=https%3A//eve.eveanalytics.com/assays/assaystudyset/53/.
