# Supplementary material for: Scaling human liver microphysiological systems: implementing a higher-throughput liver acinus microphysiological system platform
Source: Exp Biol Med (Maywood). 2026 May 22;251:11038. doi: 10.3389/ebm.2026.11038 (PMC13236629; doi:10.3389/ebm.2026.11038)
Supplement: Supplementary file 1 [file Supplementaryfile1.docx]

**Supplementary Material**

**Supplementary Methods**

**CellTracker Dye Labeling for Visualization of the LAMPS Multicellular Architecture.** Immortalized hepatocytes (Applied Biological Materials, Richmond, BC; T0054) were cultured to approximately 80% confluency, washed with 1× PBS, and incubated with 2 µM CellTracker Orange (ThermoFisher Scientific, Waltham, MA; C2927) for 15 minutes at 37°C. Following incubation, the dye-containing medium was removed, and cells were washed with warm PBS. Cells were then detached using TrypLE Express for 4 minutes, resuspended in hepatocyte replating medium, and centrifuged at 200 × g for 5 minutes. Labeled hepatocytes were subsequently loaded into the microfluidic device as described in the Methods. Liver sinusoidal endothelial cells (LSECs) were cultured to approximately 80% confluency in fibronectin-coated T-25 flasks. Cells were processed similarly to hepatocytes, except that 2 µM CellTracker Green (ThermoFisher Scientific; C2925) was used for labeling. LSECs were resuspended at a density of 3 × 10⁶ cells/mL and combined in equal volumes with THP-1 cells that had been labeled with 10 µM CellTracker Blue (ThermoFisher Scientific; C2111) at a density of 1.6 × 10⁶ cells/mL. This mixed cell suspension was loaded into the device following hepatocyte seeding and extracellular matrix overlay. LX-2 hepatic stellate cells were incubated with 0.5 µM CellTracker Deep Red (ThermoFisher Scientific; C34565) and incorporated into the collagen overlay at a density of 1.4 × 10⁵ cells/mL, as described in the Methods.

**Supplementary Figures**

**
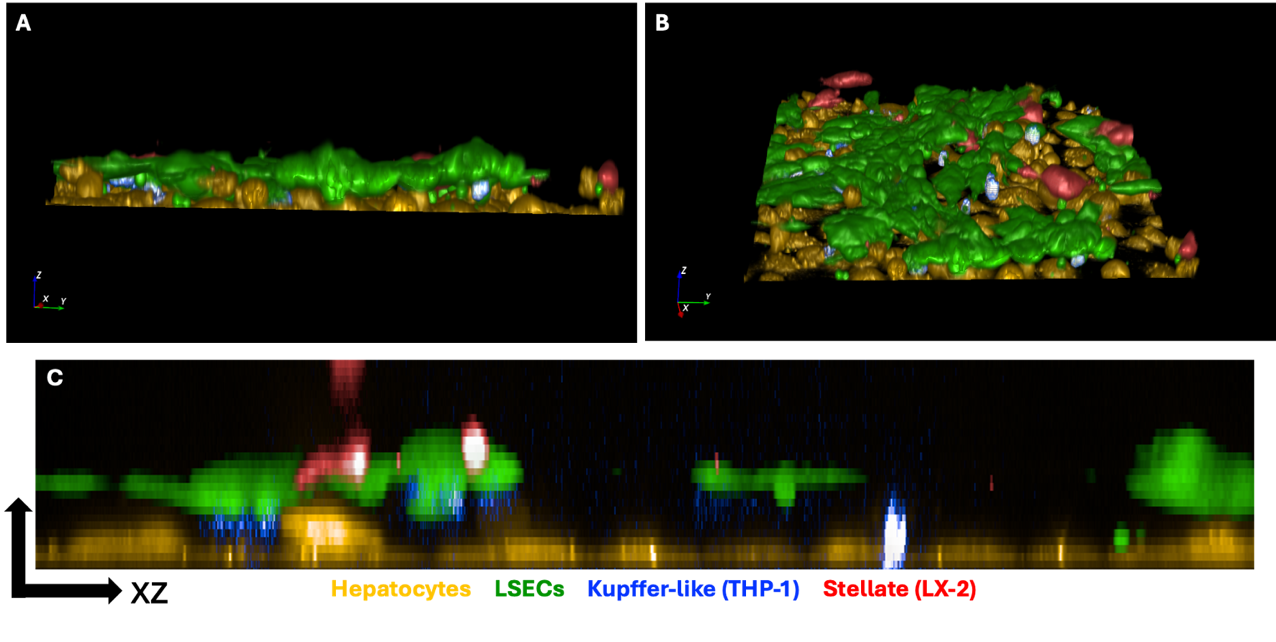
**

**Supplementary Figure 1. Three-dimensional spatial organization of the four-cell-type distribution in ht-LAMPS.** (A, B) Representative 3D renderings (40X) of ht-LAMPS under normal fasting (NF) conditions showing the spatial distribution of the four liver cell types labeled with CellTracker dyes. Hepatocytes (yellow; Ex 561 nm / Em 570–630 nm) are primarily localized near the base of the device. Liver sinusoidal endothelial cells (LSECs; green; Ex 488 nm / Em 500–550 nm) are positioned above the hepatocyte layer. THP-1–derived Kupffer-like cells (blue; Ex 405 nm / Em 435–480 nm) and hepatic stellate cells (LX-2; red; Ex 640 nm / Em 650–670 nm) are distributed throughout the construct, with the majority localized within the collagen matrix above the LSEC layer. (C) Representative XZ projection illustrating the vertical distribution of the four-cell-type architecture in ht-LAMPS. The layered organization of hepatocytes at the base, followed by LSECs, with Kupffer-like cells and stellate cells distributed within the upper regions of the MPS configuration schematic shown in Figure 1.

**
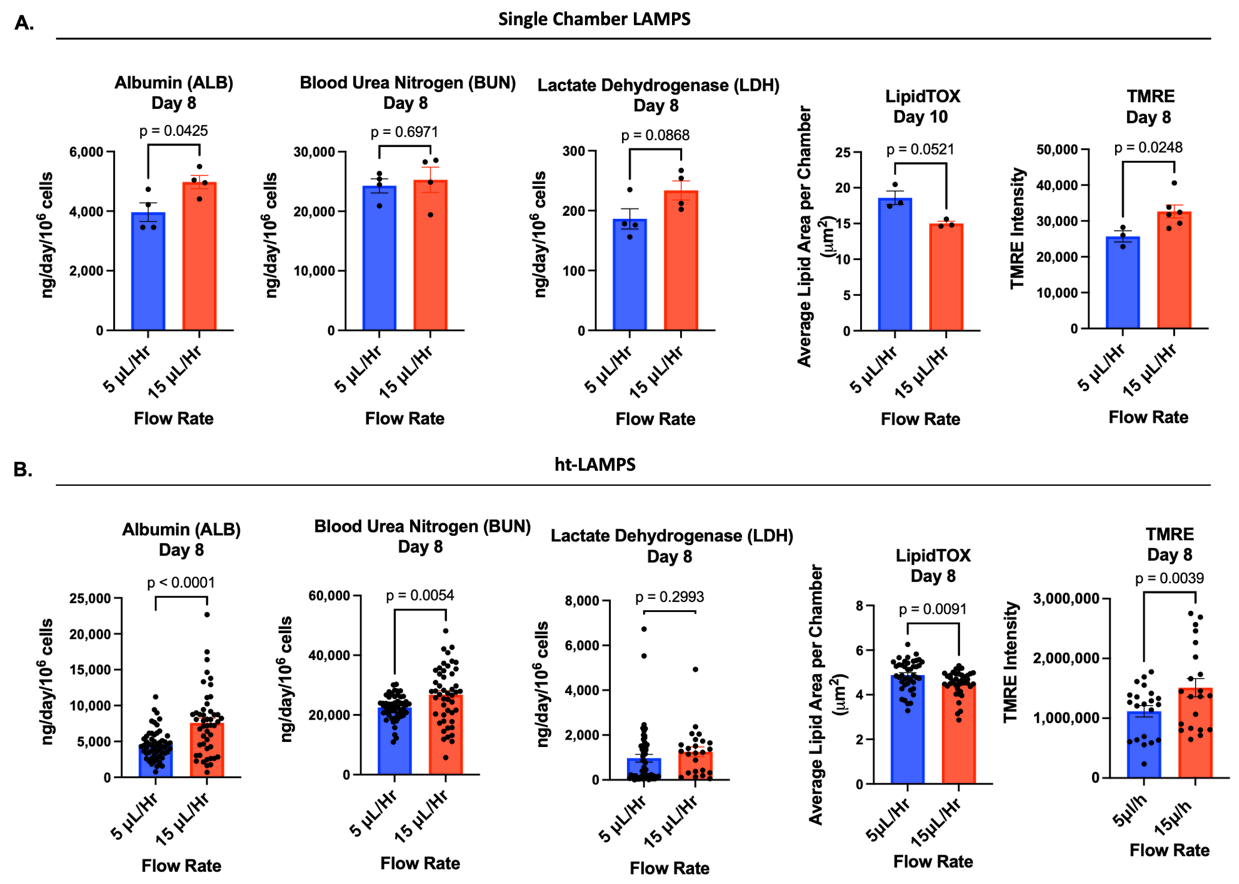
**

**Supplementary Figure 2. Graphical representation of zonation-dependent functional metrics in single-chamber LAMPS and ht-LAMPS platforms.** (A) Functional outputs measured in single chamber LAMPS under Zone 3–like and Zone 1–like flow conditions, including albumin (ALB), blood urea nitrogen (BUN), lactate dehydrogenase (LDH), steatosis (LipidTOX), and mitochondrial activity (TMRE). Data are shown for individual metrics at the indicated time points and correspond to the values used to calculate Zone 3 to Zone 1 (Z3/Z1) ratios summarized in Supplementary Table 2. (B) Corresponding functional outputs measured in ht-LAMPS under Zone 3–like and Zone 1–like flow conditions for the same metrics. Individual data points represent measurements from individual chambers, with error bars indicating mean ± SEM. Across platforms, similar directional relationships between Zone 3 and Zone 1 conditions are observed for these metrics. While these datasets were not generated under fully matched head-to-head conditions, they were derived from experiments performed separately using the same hepatocyte lot across platforms (except for TMRE), demonstrating consistent zonation-dependent patterns for the metrics evaluated.

**
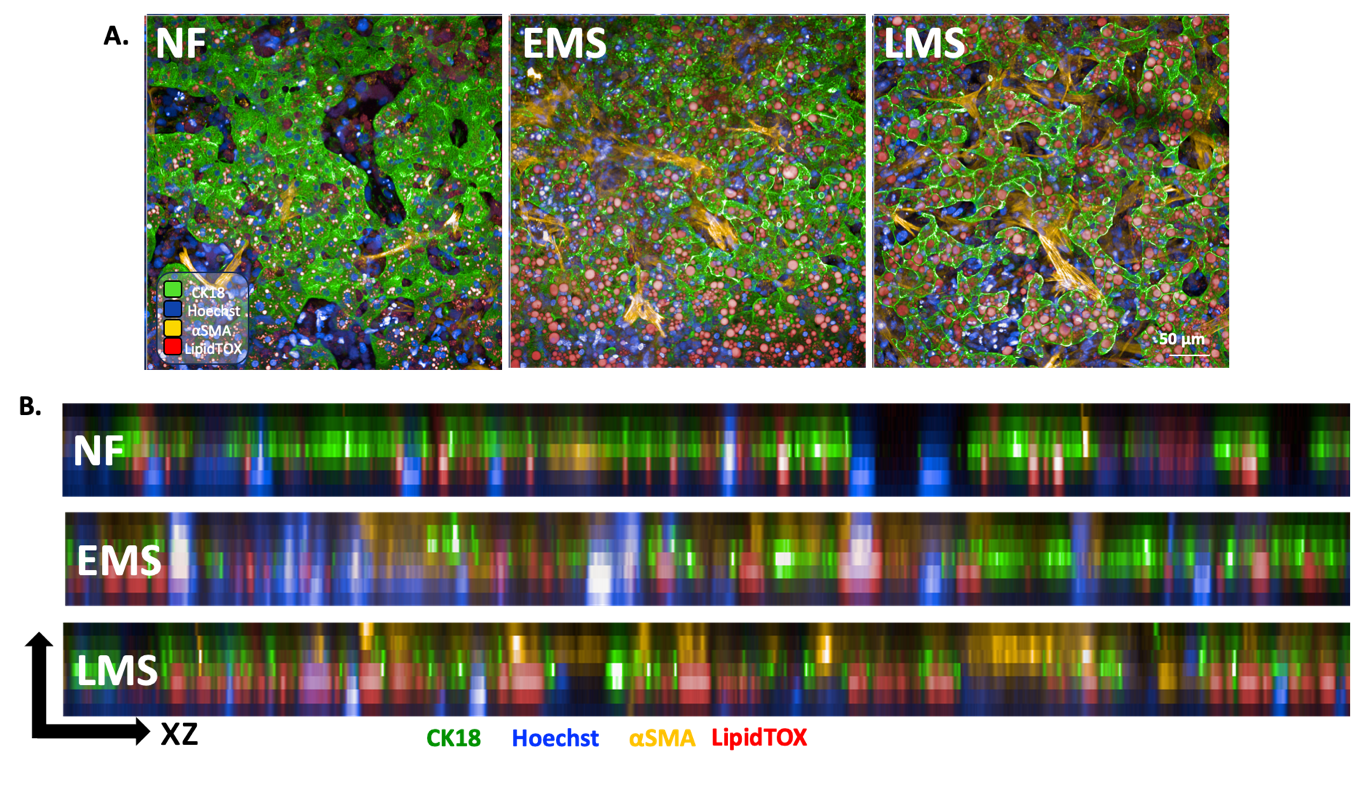
**

**Supplementary Figure 3. Spatial organization of hepatocytes and stellate cells in ht-LAMPS under MASLD-inducing conditions.** (A) Representative 20X immunofluorescence images of ht-LAMPS cultured under normal fasting (NF), early metabolic syndrome (EMS), and late metabolic syndrome (LMS) media conditions shown in Figure 3C; Scale bar = 50 µm. Hepatocytes are labeled with cytokeratin-18 (CK-18; green), nuclei with Hoechst (blue), activated stellate cells with α-smooth muscle actin (αSMA; yellow), and neutral lipid accumulation with LipidTOX (red). Increased αSMA and LipidTOX signal is observed under EMS and LMS conditions relative to NF, consistent with enhanced stellate cell–associated marker expression and lipid accumulation under disease-inducing conditions. (B) Representative XZ projections from these images illustrating the vertical distribution of cell populations and lipid accumulation within the ht-LAMPS chamber. Hepatocytes (CK-18; green) are primarily localized near the base of the chamber and co-localize with nuclei (Hoechst; blue) and LipidTOX signal (red). αSMA–positive stellate cells (yellow) are distributed above the hepatocyte layer, with increased signal intensity and apparent infiltration under EMS and LMS conditions relative to NF. These data demonstrate the spatial organization of the multicellular system and qualitative changes in cellular distribution and marker expression under MASLD-inducing media conditions.

**Supplementary Tables**

| **Supplementary Table 1.** Key components for the formulation of Normal Fasting (NF), Early Metabolic Syndrome (EMS) and Late Metabolic Syndrome (LMS) media formulations. | | | |
| --- | --- | --- | --- |
| **Medium Component** | **NF**  **(Normal Fasting)** | **EMS**  **(Early Metabolic Syndrome)** | **LMS**  **(Late Metabolic Syndrome)** |
| **Glucose** | 5.5 mM | 11.5 mM | 20 mM |
| **Insulin** | 10 pM | 10 nM | 10 nM |
| **Glucagon** | 100 pM | 30 pM | 30 pM |
| **Oleic acid** | - | 200 µM | 200 µM |
| **Palmitic acid** | - | 100 µM | 100 µM |
| **Lipopolysaccharide**  **(LPS)** | - | - | 0.25 µg/mL |
| **Transforming Growth Factor β1 (TGF-β1)** | - | - | 5 ng/mL |
| Media formulations were designed to mimic disease progression from the normal fasting (NF) to early metabolic syndrome (EMS; MASLD) and late metabolic syndrome (LMS; MASH) state. These media formulations were developed using glucose-free Williams E base medium supplemented with varying levels of glucose, insulin, glucagon, oleic acid, palmitic acid and molecular drivers of fibrosis including TGF-β1 and LPS. | | | |

| Supplementary Table 2. Zone-specific Comparison of metrics between single chamber LAMPS and ht-LAMPS platforms. | | |
| --- | --- | --- |
| **Metric (Day 8)** | **Single Chamber LAMPS**  **Z3/Z1 ratio** | **ht-LAMPS**  **Z3/Z1 ratio** |
| **Albumin** | 0.79 ± 0.08 | 0.56 ± 0.12 |
| **Urea Nitrogen** | 0.96 ± 0.16 | 0.83 ± 0.04 |
| **Lactate Dehydrogenase** | 0.80 ± 0.07 | 1.05 ± 0.41 |
| **LipidTOX** | 1.24 ± 0.03  (day 10) | 1.20 ± 0.03 |
| **TMRE*** | 0.79 ± 0.04 | 0.77 ± 0.15 |
| Single chamber LAMPS and ht-LAMPS values are displayed as the average ratio of Zone 3 to Zone 1 values on Day 8 ± SEM, unless day indicated otherwise. All Z3/Z1 comparisons were made using the same lot of human hepatocytes (hu8391), except for TMRE (*) which in the single chamber LAMPS was performed using hepatocyte lots hu8339 and hu1960. | | |

| Supplementary Table 3. Comparison of MASLD phenotypic metrics between the single chamber LAMPS and ht-LAMPS in EMS and LMS media. | | | |
| --- | --- | --- | --- |
| **MASLD Metric (Day 8)** | **Media Condition** | **Single Chamber LAMPS** | **ht-LAMPS** |
| **Steatosis** | EMS | 2.97 ± 0.12 | 1.40 ± 0.01 |
|  | LMS | 3.09 ± 0.25  (day 10) | 1.35 ± 0.04 |
| **IL-6 Secretion** | EMS | 2.50 ± 0.10 | 0.98 ± 0.32 |
|  | LMS | 4.05 ± 0.46 | 3.86 ± 0.44 |
| **IL-8 Secretion** | EMS | 1.19 ± 0.20 | 0.55 ± 0.10 |
|  | LMS | 2.66 ± 0.15 | 1.48 ± 0.24 |
| **CCL2 Secretion** | EMS | 1.50 ± 0.06 | 0.37 ± 0.12 |
|  | LMS | 3.26 ± 0.20 | 1.97 ± 0.58 |
| **COL 1A1 Secretion** | EMS | 1.03 ± 0.02 | 2.44 ± 0.14 |
|  | LMS | 2.38 ± 0.13 | 3.15 ± 0.36 |
| Single chamber LAMPS and ht-LAMPS values are displayed as the average change normalized to NF medium values on Day 8, unless day indicated otherwise. | | | |
